# The Stigmatizing Attitudes of Syrian University Students Toward Schizophrenia

**DOI:** 10.7759/cureus.29504

**Published:** 2022-09-23

**Authors:** Sarya Swed, Sheikh Shoib, Saeed Kadri, Noheir A Hassan, Abdulqadir J Nashwan, Mohammad B Almoshantaf, Tasneem Mohamed, Bisher Sawaf, Nashaat K Elkalagi, Amine Rakab

**Affiliations:** 1 Department of Medicine, Aleppo University, Aleppo, SYR; 2 Department of Psychiatry, Government Medical College, Srinagar, IND; 3 Faculty of Medicine, Syrian Private University, Damascus, SYR; 4 Faculty of Medicine, Aswan University, Aswan, EGY; 5 QU (Qatar University) Health, Qatar University, Doha, QAT; 6 Department of Nursing, University of Calgary In Qatar, Doha, QAT; 7 Department of Nursing Education, Hamad Medical Corporation, Doha, QAT; 8 Department of Neurosurgery, Ibn Al-Nafees Hospital, Damascus, SYR; 9 Department of Neurosurgery, National Ribat University, Khartoum, SDN; 10 Department of Internal Medicine, Faculty of Medicine, Syrian Private University, Damascus, SYR; 11 Faculty of Medicine, Al Arish University, Al Arish, EGY; 12 Department of Internal Medicine, Weill Cornell Medicine – Qatar, Doha, QAT

**Keywords:** medical and non-medical students, gender differences, college students, social distance, stigma, schizophrenia

## Abstract

Background: Stigma is frequently considered an obstacle to schizophrenia treatment and recovery. However, little is known regarding the stigma experienced by persons with schizophrenia among Syrian college students.

Methods: A total of 963 students from Syrian colleges and universities participated in this study, using a questionnaire with a case vignette illustrating schizophrenia. The questionnaire inquired about people's attitudes toward schizophrenia and their desire to avoid contact with people with schizophrenia. The current study investigated college students' stigma toward people with schizophrenia, desire for social distancing, gender (male and female), and major (medical and non-medical) differences.

Results: The people described in the vignette were deemed "dangerous" (28%) and "could snap out of the problem" (50.20%), according to the respondents. Female students were more likely than male students to agree that "schizophrenia is not really a medical disease" (4.40% vs. 8.9%, p ≤ 0.05). Compared with medical students, non-medical students were more likely to agree that "The problem is a reflection of personal weakness" (20% vs. 21.7%, p < 0.05). Many respondents said they would not "marry into the family of someone with schizophrenia" (95.6%) or "work closely with them" (77.6%).

Conclusion: In this study, it was observed that a considerable percentage of Syrian college students exhibited stigma toward persons with schizophrenia and really wanted to avoid social interaction with them, with female and non-medical students having higher stigma toward people with schizophrenia in several subscale items. The findings imply that more anti-stigma interventions for Syrian college students should be implemented to help avoid or lessen the stigma toward people with schizophrenia.

## Introduction

The stigma associated with mental illness is pervasive. Patients who suffer from mental illness are isolated because of a lack of understanding of the actual nature of the condition among others who stigmatize them and choose not to interact with them [1]. The majority of people who suffer from mental illness face many difficulties. First, they are dealing with the symptoms of their sickness. On the other hand, they suffer from the feeling of social rejection as a result of the general public's ignorance of the nature of the mental illness. Two major types of stigmas are public stigma and self-stigma. Public stigma is the reaction of the population to mentally ill people. Self-stigma is the internalized shame mentally ill people have about their condition [2]. Most mentally ill people experience negative attitudes from others, such as avoidance, forcing them to seek treatment, isolating them from others, and hostile behavior [3]. 

They may experience social exclusion and isolation, affecting their family relationships badly [4]. People with mental illnesses experience stigmatizing views and seek assistance for mental health concerns; Canadian research reveals that over 70% of people with mental illnesses do not obtain any treatment [5]. This is because stigma and discrimination against people diagnosed with mental illness or those who receive treatment for their mental illness act as a barrier to seeking healthcare programs, causing more deterioration of their condition [6].

Research has also noticed a wide mortality gap between mentally ill people and the general population [7]. A higher risk of suicide can partially explain this high mortality rate among people with mental illness among the most vulnerable groups [8]. Cardiovascular, accidental, respiratory, and cancer-related fatalities are also known to be significant causes of mortality among people with mental health disorders. Anti-stigma initiatives have been set up to tackle the stigmatizing attitudes against mental illness and discriminating actions among individuals and society [9]. Schizophrenia is a severe mental illness that has been described as "the worst disease affecting humanity"; it is one of the top 10 leading causes of disease-related disability globally [10]. However, its etiology is still unknown. Several genetic and environmental factors may have contributed to its development [11].

A study from the UK [12] showed that university students have little understanding of schizophrenia, and stigma is common. Researchers have discovered that stigmatizing sentiments persist in the broader public, especially among students [13-16]. Our study was meant to find out how common public stigma about schizophrenia, including personal and perceived stigma, was among a group of Syrian college students and what caused it. 

## Materials and methods

Sample

This was a cross-sectional study. Data were collected from an Online Google Form published on social media from 18th to 27th March 2021. An online tool [17] was used to calculate the minimum sample size in our study. According to the data from the United Nations, the estimated number of the Syrian population is about 18 million in 2019 [18]. The required sample size appeared to be 385 participants. The sample size was calculated as the proportion of the stigma to 50%, at the 95% confidence level with a 5% marginal error. The total number of participants who completed the online questionnaire was 936, with one who refused to complete it. The convenience sampling method was used in the present study. Considering the sample's representativeness, this study randomly selected different classes by school, grade, and major. We included Syrian university students who are over 18 and live in Syria, but we excluded others.

Measurements in the survey questionnaires

The questionnaire was adapted from many published papers in the existing literature [19-23]. The survey was translated from the English language to the Arabic language by an expert in the translation field to generate an obvious and clear questionnaire for the participants, as well we assess the validity of the utilized subscales (personal stigma toward schizophrenia scale, personal stigma toward schizophrenia scale, and social distance scale [SDS]) by conducting a pilot study on 35 participants to calculate the Cronbach's alpha of them, which was above 0.7 for all.

The questionnaire consisted of six sections as follows:

First section: Included a range of questions about demographic variables such as age, gender, and social status.

Second section: Included nine questions about personal stigma toward schizophrenia scale and nine questions about personal stigma toward schizophrenia scale [24]. In the personal stigma subscales, respondents were asked about their attitude toward people with schizophrenia symptoms described in the vignette (e.g., “People with schizophrenia could snap out of it if they wanted”). In the perceived stigma subscales, respondents were asked their beliefs about most of the other people’s attitudes toward people with schizophrenia symptoms described in the vignette (e.g., “Most people believe that people with schizophrenia could snap out of it if they wanted”). The response of each item was measured on a five-point scale ranging from “strongly agree” to “strongly disagree.” 

Third section: Included close-ended questions about social distance with those with schizophrenia [25]. The response of each item was measured on a four-point scale, which ranged from “definitely willing” to “definitely unwilling.” The reliability and validity of its Chinese version have been tested, and the results showed that all the indicators met the requirements of psychometrics.

Fourth section: Consisted of questions about the participants' usual sources of knowledge about schizophrenia-like newspapers, TV, or websites.

Fifth section: It was concerned with helpfulness or intervention; this part was subdivided into four subgroups of questions with multiple answers; these subgroups are people who can help, medications that can help, and other interventions and help methods [23].

Sixth section: It was concerned with supporting information to assess participants' knowledge to define the correct diagnosis for each vignette, which was taken from another research [26]; it included three vignettes describing a person with schizophrenia, depression, or generalized anxiety disorder.

Ethics statement

The protocol was approved by the joint ethics committee of Damascus University and Aleppo University in March 2021 (Reference number: 225). The aim of the present study was explained in the questionnaires, and informed consent was obtained from all the respondents through a Yes-or-No question inside the questionnaire asking participants whether they agreed to answer this questionnaire or not. They were encouraged to independently analyze the vignette and answer several questions, including demographic information, schizophrenia stigma scale, and SDS. The survey contained a cover page stating that responses were anonymous and voluntary and would not impact the participants. All methods were performed in accordance with the relevant guidelines and regulations of the declaration of Helsinki.

Statistical analysis

The Statistical Package for the Social Sciences (SPSS 22; IBM Corp., Armonk, NY) and Microsoft Excel. Descriptive statistics were applied for demographic data (percentage), stigmatizing attitudes toward schizophrenia patients (percentage frequencies and 95% CI), and social spacing (percentage frequencies and 95% CI). On the schizophrenia stigma scale, the categories "agree" and "strongly agree" were included in one option, and on the SDS, the categories “Yes” and “No” were also included in one option. The combined selections indicate the respondents' positive and negative sentiments. The significant difference in each item on the schizophrenia stigma scale and SDS among various demographic characteristics were assessed using Pearson's chi-square test (gender, major, educational level, and school level) in the proportion of agreement. The value of p was set at <0.05 for statistical significance.

## Results

Demographic baseline characteristics of the study sample

Among the 936 handed-out questionnaires, just one declined to complete it, so the final number of responses that were eligible for statistical analysis was 935. Respondents' general characteristics are shown in Table 1. The respondents' average age was 22.8±4.37 years (mean ± SD). The gender ratio was roughly 1:2 (male 33.6% : female 66.4%).To investigate the differences in stigmatizing demeanor between those two groups, we divided student majors into medical (62.5%) and non-medical (37.5%). Approximately 34.8% of individuals worked during their school years. There is a convergence of percentages between those who have reported positive and negative histories of mental health disease, where the percentage of the sufferers is 49.3%. However, only 4.8% received psychological treatment.

**Table 1:** Baseline characteristics of the participants

Variable	Options	N=935	
		n	%
Age	(Mean/SD)	22.8/4.37	
Gender	Male	314	33.6%
	Female	621	66.4%
Social status	Single	831	88.9%
	Married	93	9.9%
	Divorced	9	1.0%
	Widower	2	0.2%
Major section	Medical student	584	62.5%
	Non-medical student	351	37.5%
Economic level	Low	60	6.4%
	Middle	524	56.0%
	Good	304	32.5%
	High	47	5.0%
The university stages	First year	154	16.5%
	Second year	128	13.7%
	Third year	132	14.1%
	Fourth year	177	18.9%
	Fifth year	159	17.0%
	Sixth year	185	19.8%
Region	Urban	672	71.9%
	Rural	263	28.1%
Occupation status	Employed	325	34.8%
	Unemployed	610	65.2%
Living with	Family	796	85.1%
	Father	12	1.3%
	Mother	56	6.0%
	Friends	71	7.6%
Immigrant status	Yes	341	36.5%
	No	594	63.5%
History of mental health disease	Yes	461	49.3%
	No	474	50.7%
Current psychological treatment	Yes	45	4.8%
	No	890	95.2%
Current pharmacological treatment	Yes	138	14.8%
	No	797	85.2%

**Figure 1:**
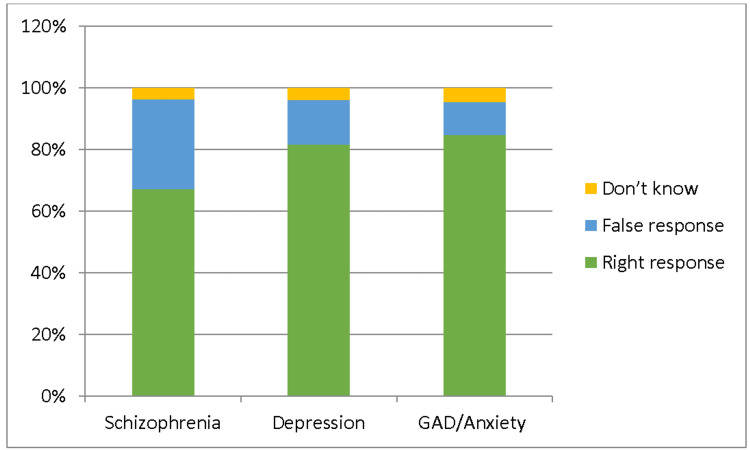
Participants’ responses about schizophrenia GAD: Generalized anxiety disorder

We found high percentages of correct knowledge responses when diagnosing three mental diseases. The percentages of incorrect replies in the absence of diagnostic anxiety, depression, and schizophrenia were 9.4%, 10.5%, and 20.3%, respectively. Other responses are shown in Figure 1.

Personal stigma

Table 2 depicts gender differences and medical and non-medical inequalities in the percentage of individuals who held stigmatizing attitudes toward people with schizophrenia. Approximately 47.8% of respondents, the bulk of whom were females, stated that schizophrenic people might snap out of it. Only 14.6% of respondents acknowledged that they believe schizophrenia to be a real medical illness. Interestingly, 2% of respondents with a medical background believed the same thing. Regarding more severe social stigma, 16.80% of respondents believed schizophrenia persons were severe. Twenty-five percent of persons without a medical background will avoid contact with those who have schizophrenia, and that number rises to 5.2% among those who do. About 18.8% of those polled claimed they would not inform anybody if they were diagnosed with schizophrenia. Only a small portion of respondents (6.9%) said they would not vote for or hire a candidate with schizophrenia (8.4%).

**Table 2:** Percentage of participants who “agree” or “strongly agree” about personal stigma toward schizophrenia patient scale statements CI, confidence interval; SPSS, schizophrenia personal stigma scale. Data are n, %, or mean ± SD. **p < 0.001, *p < 0.05.

Statement about personal belief	Total (N=935)		Gender				Major section				Region				Economic level										Occupation status				
	n		Male (n=314)		Female (n=621)		Medical (n=584)		Non-medical (n=351)		City (n=672)		Rural region (n=263)		Low (n=60)		Moderate (n=524)			Good (n=304)		High (n=47)			Worker (n=325)			Non-worker (n=610)	
		%																												n	%	n	%	n	%	n	%	n	%	n	%	n	%		n	%	n	%		n	%		n	%	n	%
		The person could snap out of schizophrenia																												602	47.80%	115	9.20%	477	38.2%**	179	14.30%	413	33.1%**	421	33.70%	171	13.70%	37	3.00%		370	29.60%	171	13.70%		14	1.1%*		247	19.80%	345	27.60%
		Schizophrenia is a sign of personal weakness	149	11.80%	48	3.90%	86	6.9%*	30	8.40%	104	2.4%**	80	6.40%	54	4.3%*	17	8.40%		77	6.20%	35	2.80%		5	0.40%		62	5.00%	72	5.85
Schizophrenia is not a real medical illness	184	14.60%	55	4.40%	111	8.9%*	25	2.00%	141	11.4%**	101	8.10%	65	5.2%*	22	1.80%		107	8.60%	34	2.70%		3	0.2%*		72	7.60%	94	5.80%
People with schizophrenia are dangerous	211	16.80%	57	4.60%	154	12.40%	74	5.90%	137	11.00%	148	11.90%	63	5.10%	19	1.50%		130	10.50%	53	4.30%		9	0.70%		97	9.20%	114	7.80%
I avoid people with schizophrenia	245	19.50%	56	4.50%	189	15.20%	65	5.20%	180	14.5%**	179	14.40%	66	5.30%	24	1.90%		139	11.20%	76	6.10%		6	0.50%		120	9.70%	125	10.15
People with schizophrenia are unpredictable	399	31.70%	91	7.40%	308	25%*	127	10.30%	272	22.10%	287	23.30%	112	9.10%	37	3.00%		241	19.50%	115	9.30%		6	0.5%*		157	12.70%	242	19.6%*
If I had schizophrenia, I would not tell anyone	237	18.80%	71	5.70%	166	13.40%	63	5.10%	174	14%**	171	13.80%	66	5.30%	24	1.90%		142	11.40%	59	4.80%		12	1.00%		98	11.20%	139	7.90%
I would not employ someone with schizophrenia	87	6.90%	27	2.20%	60	4.80%	24	1.90%	63	5.10%	65	5.20%	22	1.80%	15	1.20%		47	3.80%	19	1.50%		6	0.5%*		42	3.40%	48	3.60%
I would not vote for a politician with schizophrenia	106	8.40%	33	2.70%	73	5.90%	38	3.10%	68	5.5%**	75	6.00%	31	2.50%	9	0.70%		58	4.70%	32	2.60%		7	0.60%		53	4.30%	53	4.30%
SPSS total score (mean ± SD)	1.74	1.4	1.6	1.4	1.7	1.4	1.4	1.2	1.8	1.5	1.7	1.4	1.7	1.4	1.9	1.5		1.7	1.4	1.6	1.3		1.6	1.7		1.7	1.4	1.7	1.4

Perceived stigma

Table 3 displays the degree of agreement among participants on issues that gauged the views of other participants toward schizophrenic people. In line with half of the participants, most people assume people with schizophrenia can break out of it if they want it. When comparing perceived and personal stigma (Tables 2, 3), approximately 37.8% of respondents believed that schizophrenia is a sign of weakness. However, only 11.8% of respondents genuinely believed this when expressing their personal beliefs on the subject. According to 25% of non-medical participants, most people do not really regard schizophrenia to be a medical condition, and 17.7% believed that schizophrenic persons were dangerous. According to 38.6% of respondents, the majority of individuals believed it was better to avoid spending time with persons who have schizophrenia in order to avoid developing the disease themselves. Furthermore, 31.9% of participants believed that most people would not hire or vote for someone with schizophrenia.

**Table 3:** Percentage of participants who "agree" or "strongly agree" with perceived stigma toward schizophrenia patient scale statements CI, confidence interval; SPSS, schizophrenia perceived stigma scale. Data are n, %, or mean ± SD. **p < 0.001, *p < 0.05.

Statement about perceived belief	Total (N=935)		Gender				Major section				Region				Economic level										Occupation status				
	n		Male (n=314)		Female (n=621)		Medical (n=584)		Non-medical (n=351)		City (n=672)		Rural region (n=263)		Low (n=60)		Moderate (n=524)			Good (n=304)		High (n=47)			Worker (n=325)			Non-worker (n=610)	
		%																												n	%	n	%	n	%	n	%	n	%	n	%	n	%		n	%	n	%		n	%		n	%	n	%
		Most people believe that people with schizophrenia could snap out of it if they wanted																												632	50.20%	132	11.30%	500	42.9%**	235	20.20%	397	34.00%	452	38.80%	180	15.40%	51	4.40%		376	32.20%	187	16.00%		18	1.50%		273	23.40%	359	30.80%
		Most people believe that schizophrenia is a sign of personal weakness	476	37.80%	107	9.40%	369	32.3%*	228	20.00%	248	21.7%**	356	31.20%	120	10.5%*	36	3.20%		295	25.90%	128	11.20%		17	1.50%		207	18.10%	269	23.60%
Most people believe that schizophrenia is not a medical illness	518	41.10%	118	10.50%	400	35.5%*	231	20.50%	287	25.5%**	375	33.30%	143	12.70%	41	3.60%		313	27.80%	146	13.00%		18	1.60%		229	20.30%	289	25.60%
Most people believe that people with schizophrenia are dangerous	353	28.00%	74	6.60%	279	24.8%**	154	13.70%	199	17.7%**	257	22.90%	96	8.50%	27	2.40%		211	18.80%	101	9.00%		14	1.20%		150	13.40%	203	18.10%
Most people believe that it is best to avoid people with schizophrenia so that you don't become depressed yourself	486	38.60%	99	8.80%	387	34.5%**	213	19.00%	273	24.3%**	364	32.40%	122	10.9%*	34	3.00%		284	25.30%	155	13.80%		13	1.20%		209	18.60%	277	24.70%
Most people believe that people with schizophrenia are unpredictable	361	28.70%	84	7.50%	277	24.9%*	124	11.10%	237	21.3%**	254	22.80%	107	9.60%	29	2.60%		221	19.80%	98	8.80%		13	1.20%		162	14.50%	199	17.90%
If they had schizophrenia, most people would not tell anyone	513	40.70%	113	10.10%	400	35.8%**	216	19.30%	297	26.6%**	371	33.20%	142	12.75	37	3.30%		306	27.40%	153	13.70%		17	1.50%		220	19.70%	293	26.20%
Most people would not employ someone they knew had been affected by Schizophrenia	402	31.90%	84	7.50%	318	28.4%**	186	16.60%	216	19.3%**	300	26.80%	102	9.1%*	30	2.70%		234	20.90%	122	10.90%		16	1.40%		179	16.00%	223	19.90%
Most people would not vote for a politician they knew had been affected by Schizophrenia	401	31.90%	79	7.10%	322	28.8%**	178	15.90%	223	19.9%**	289	25.80%	112	10.00%	33	3.00%		235	21.00%	118	10.60%		15	1.30%		178	15.90%	223	19.90%
SPSS total score (mean±SD)	3.7	2.90	2.9	2.70	3.9	2.9**	4.2	3	3.3	2.7**	3.8	2.9	3.4	2.8	3.4	2.9		3.7	2.8	3.6	2.9		3.4	3.4		3.6	2.8	3.7	2.8

Social distance

The percentages of participants who said they were "probably unwilling" or "definitely unwilling" to have interaction with schizophrenic patients are listed in Table 4. Generally, more than 80% of participants would not live next door to people with schizophrenia if given the option. More than half (54%) and almost all (95.6%) would not want to associate with or marry one of them.

**Table 4:** Percentage of participants who were "probably unwilling" or "definitely unwilling" to have contact with schizophrenic patients CI, confidence interval; SDS, social distance scale. Data are n, %, or mean ± SD. **p < 0.001, *p < 0.05.

Statement about personal belief (SDS	Total (N=935)		Gender				Major section				Region				Economic level										Occupation status				
	n		Male (n=314)		Female (n=621)		Medical (n=584)		Non-medical (n=351)		City (n=672)		Rural region (n=263)		Low (n=60)		Moderate (n=524)			Good (n=304)		High (n=47)			Worker (n=325)			Non-worker (n=610)	
		%																												n	%	n	%	n	%	n	%	n	%	n	%	n	%		n	%	n	%		n	%		n	%	n	%
		Live next door																												751	80.30%	261	28.30%	490	53.10%	461	49.90%	290	31.40%	538	58.30%	213	23.10%	42	4.60%		429	46.50%	243	26.30%		37	4.00%		261	28.30%	490	53.10%
		Spend the evening socializing	505	54.00%	182	19.60%	323	34.80%	353	38.00%	152	16.4%**	357	38.50%	148	15.90%	33	3.60%		280	30.20%	170	18.30%		22	2.40%		176	19%	329	35.50%
Make friends	608	65.00%	214	23.20%	394	42.70%	382	41.40%	226	24.50%	435	47.10%	173	18.70%	40	4.30%		340	36.80%	198	21.50%		30	3.30%		206	22.30%	402	43.60%
Work closely	726	77.60%	248	26.80%	478	51.60%	447	48.20%	279	30.10%	519	56.00%	207	22.30%	39	4.20%		416	44.90%	233	25.10%		38	4.10%		242	26.1%*	484	52.20%
Marry into family	894	95.60%	301	32.50%	593	64.00%	557	60.10%	337	36.40%	639	65.90%	256	20.50%	57	6.10%		501	54.00%	295	31.80%		41	4.4%*		313	33.8%	581	62.70%
SDS total score (mean±SD)	3.7664	1.26	3.87	1.21	3.709	1.28*	3.803	1.29	3.70	1.21	3.74	1.28	3.81	1.21	3.51	1.32		3.79	1.23	3.79	1.27		3.60	1.48		3.80	1.25	3.69	1.27

Participants’ usual sources of mental health knowledge

**Figure 2:**
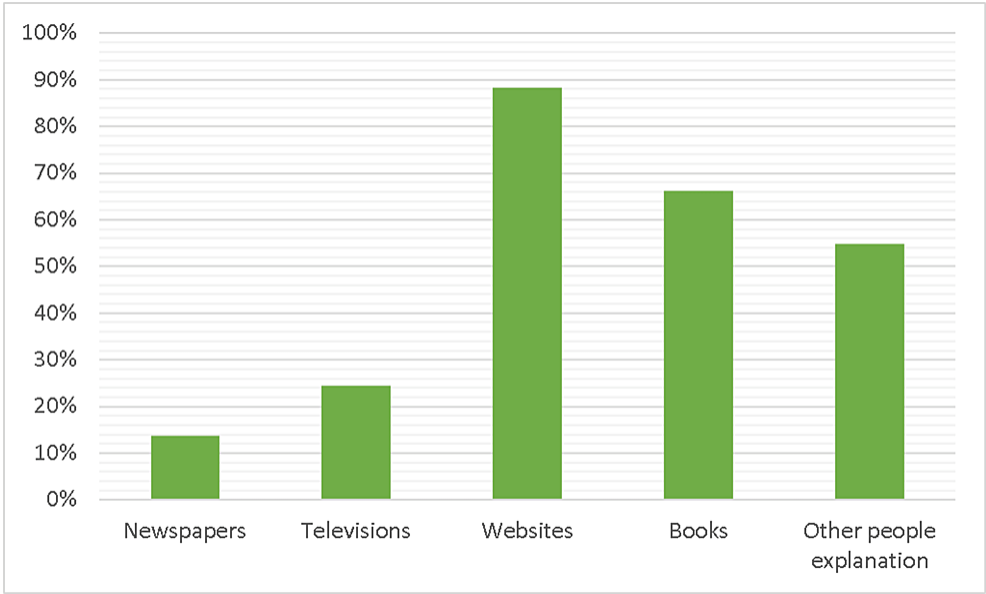
Supporting Information (sources of knowledge about schizophrenia)

Figure 2 shows that online websites were the most reliable source of information for mental health understanding (84.5%). Books (72.1%), people's explanations (50.20%), television (20.7%), and newspapers (12.60%) were all considered trustworthy sources of knowledge.

Helpful individuals and interventions

Table 5 contains questions about individuals, treatment, and other interventions that respondents find helpful for people with schizophrenia. According to 87.4% of participants, a psychiatrist would greatly help with such mental health concerns. About 58% said enlisting a close relative's help would be advantageous, and 45.9% agreed that praying to God would be beneficial. Antidepressants (69.4%), anxiolytics (60.3%), and antipsychotics (57.8%) were the three leading prescriptions chosen and believed to be the most beneficial by respondents. The encouragement of psychotherapy was chosen as the most helpful intervention out of all the activities indicated to help people with schizophrenia (76.2%). However, most respondents did not believe that acupuncture therapy would provide significant benefits (3.7%).

**Table 5:** Helpful interventions

	Number	Percentage
People who can help		
A typical GP or family doctor	507	54.5%
A pharmacist	141	15.2%
A counselor	225	24.2%
A social worker	242	26%
A telephone counseling service	64	6.9%
A psychiatrist	813	87.4%
A psychiatric nurse	343	36.9%
A clinical psychologist	647	69.6%
Help from close family	539	58%
Help from close friends	536	57.6%
An herbalist	25	2.7%
Pray to God for help	427	45.9%
Medication that can help		
Vitamins and mineral	227	31.6%
Laxatives such as lactulose or Senna	18	2.1%
Tonics or herbal medicines	95	10.8%
Antibiotics	28	3.2%
Antidepressants	608	69.4%
Pain relievers such as aspirin or acetaminophen	57	6.5%
Sleeping pills	124	14.2%
Antipsychotics	506	57.8%
Tranquilizer such as diazepam	418	47.7%
Anxiolytics	528	60.3%
Other Inventions		
Becoming physically more active, such as playing more sports, or doing a lot more walking or gardening	620	68.1%
Undergoing electro-convulsive therapy	93	10.2%
Get out more	426	46.8%
Staying at home and resting	102	11.2%
Having an occasional alcoholic drink to relax	40	4.4%
Psychotherapy	693	76.2%
Attending courses on relaxation, stress management, meditation, or yoga	382	42%
Cutting out alcohol altogether	453	49.8%
Massage to relax	196	21.5%
Acupuncture therapy	34	3.7%
Being admitted to a psychiatric hospital	264	29%
Reading about people with similar problems and how they have dealt with them	512	56.3%
Going on a special diet or avoiding certain foods	200	22%
Aromatic therapy	48	5.3%
Hypnosis	109	12%
Being admitted to a psychiatric ward or general hospital	222	24.4%
Help methods		
Encourage the person to seek help	597	65.7%
Accompany the person with professional help	520	57.2%
Contact professional help on the person`s behalf	155	17.1%
Listen to the person	391	43%
Encourage the person to see a community physician	258	28.4%
Encourage the person to see a counselor	253	27.8%
Encourage the person to see a psychiatrist	697	76.7%
Give advice	346	38.1%
Encourage the person to go to the hospital	193	21.2%
Encourage the person to see a psychologist	598	65.8%
Encourage the person to go to a mental health clinic	277	30.5%
Ask if the person wants help	408	44.9%
Assess the problem/risk of harm	215	23.7%
Do an intervention	70	7.7%
Cheer the person up/boost the person`s confidence	552	60.7%
Tell the person`s parents or family	364	40%
Seek information about the person	407	44.8%
Help the person make new friends	378	41.6%
Help with chores/work	186	20.5%
Provide general support (e.g., practical, emotional)	401	44.1%
Spend time/socialize with the person	403	44.3%
Encourage the person to become physically active	433	47.6%

## Discussion

This study aimed to identify stigmatization attitudes regarding people with schizophrenia among medical and non-medical university and college students. According to our knowledge, this is the first study to use a case vignette to analyze the differences in Syrian students' stigma attitudes toward people with schizophrenia and their desire for social isolation depending on their gender (male and female), major (medical and non-medical), region (rural and city), occupation (worker and non-worker), and economic status (low, moderate, reasonable, and high). Almost all of our sample study were females (66.4%), singles (88.9%), and unemployed (65.2%). According to the study, females were more likely than males to have personal stigmatizing attitudes toward schizophrenia and maintain social distance from people with schizophrenia; as well we found high levels of stigma and a desire for social distance toward people with schizophrenia among Syrian students. Also, non-medical students were more likely than medical students to have perceived stigmatizing attitudes about schizophrenia.

According to Ka Fai Chung et al. [27], their study revealed that almost participants disagreed with contacting schizophrenia patients, keeping a distance from a person with symptoms of schizophrenia, and the negative stereotypes about schizophrenia, also less stigmatization-related attitudes were expressed by the students who had prior interaction with mentally ill people. However, our study revealed that 38.60% of respondents said that the majority of people feel it is advisable to avoid persons with schizophrenia in order to prevent being schizophrenic. In the present study, the percentage of the respondents who agreed that "schizophrenic people are hazardous" was lower (17.7%) compared to another study by Angermeyer et al. [28] that showed that 34% of the respondents agreed that the perception of dangerousness is associated with schizophrenia, and the highest percentage was in another study in São Paulo [29], where people with schizophrenia were perceived as potentially dangerous by 74.2% of interviewees. In addition, 59.0% of the sample perceived them as capable of arousing negative reactions, and 57.2% as capable of arousing discrimination in society [29].

The present results of our research might be attributed to the dismal state of insufficient mental health assistance for all Syrians, particularly mental health patients, as a result of the 11-year civil war, which has destroyed many hospitals and encouraged professionals to migrate in search of a better livelihood. Therefore, there are no ethical rules for dealing with patients with mental health issues, and insufficient healthy surveillance and care for these people [30-32].

More than 80% of participants would not live next door to people with schizophrenia if given the option. More specifically, respondents agreed that they would not work closely with them. Fifty-four percent and 95.6% would not socialize with or marry one of them. This is consistent with other studies, such as Angermeyer et al. [28], in which 54.3% of respondents said they would not marry someone with schizophrenia, and 20.5% said they would not work closely with them. The cause of keeping social distance may be due to the belief that schizophrenic people are dangerous [28]. 

Most of the respondents (87.4%) believe that mentally ill people would gain great assistance from psychiatrists, which is in line with other studies such as Holzinger et al. [33], in which most of the people who answered said they would go to a professional first.

Ibrahim et al. [34] also found that most students with mental health problems go to non-medical people like family and friends first due to the stigma felt by others. This indicates that social support is a huge step in achieving professional support in the future. Ozmen et al. [35] found that respondents agreed that psychological counseling and social support for mentally ill patients are more effective than treatment with potentially harmful and addictive medications, which is in line with our study. Avoiding professional help is likely due to self-stigma, as it may be a barrier to mental health help-seeking [35].

According to other studies that discuss the helpful intervention to help schizophrenia patients, Palou et al. [15] found that their attitudes toward mentally ill patients improved over the training course of undergraduate nursing students in the mental health field. It was also found that education about psychiatry, including how to deal with stigma, is linked to better knowledge and a more positive view of people with mental health problems. However, Altindag et al. [36] looked into the impact of anti-stigma programs, including education and contact, etc., on medical students' views about people with schizophrenia. The anti-stigma program was implemented with first-year medical students, and their attitudes toward those with schizophrenia were measured before and after the program. After one month, the assessment was repeated. Positive attitudinal improvements were observed in terms of "belief in the etiology of schizophrenia," "social spacing among Schizophrenia ill patients, care and management of them." The findings demonstrate that this program can positively influence people's views regarding schizophrenia, implying that it can be implemented in the general community. Short programs appeal because they can have a greater impact on stigmatizing attitudes with less effort and are more accessible to many individuals. It is important to routinely participate in anti-stigma activities, including education, contact, and the use of visual aids so that persons with schizophrenia may see how views change over time.

Limitations

The study limitations included the small sample size. The study's cross-sectional design does not allow the follow-up to observe the change in beliefs toward the mentally ill patient over time. On the other hand, it included only the students, so the results cannot be generalizable, and not all the socioeconomic characteristics of the participants were considered. 

## Conclusions

The development of anti-stigma programs in Syria, taking into account sociodemographic and cultural inequalities in attitudes toward people with mental disorders, is advised. These programs might include legislative and policy change, protest and advocacy, peer services, contact, mental health literacy campaigns, and education. Special focus should be paid to awareness campaigns that have been shown to be effective in helping mental health sufferers get the care they need. Programs that have been shown to have a positive impact should be given special attention. The proper portrayal of persons with schizophrenia can be explained through public education and diagnostic communication. However, the stigmatizing attitudes were investigated in the context of hypothetical scenarios, and no causal conclusions could be drawn because the study was cross-sectional, not all social characteristics of the Syrian population were taken into account, and the study's population was limited to students, so the findings could not be generalized.
